# The Prediction of Hepatitis E through Ensemble Learning

**DOI:** 10.3390/ijerph18010159

**Published:** 2020-12-28

**Authors:** Tu Peng, Xiaoya Chen, Ming Wan, Lizhu Jin, Xiaofeng Wang, Xuejie Du, Hui Ge, Xu Yang

**Affiliations:** 1 School of Computer Science and Technology, Beijing Institute of Technology, Beijing 100081, China; pengtu@bit.edu.cn (T.P.); 3220180788@bit.edu.cn (X.C.); 2 Chinese Center for Disease Control and Prevention, Beijing 102206, China; wangming@chinacdc.cn (M.W.); jinlz@chinacdc.cn (L.J.); wangxf@chinacdc.cn (X.W.); duxj@chinacdc.cn (X.D.)

**Keywords:** hepatitis E, ensemble learning, prediction

## Abstract

According to the World Health Organization, about 20 million people are infected with Hepatitis E every year. In 2015, there were 44,000 deaths due to HEV infection worldwide. Food, water and climate are key factors that affect the outbreak of Hepatitis E. This paper presents an ensemble learning model for Hepatitis E prediction by studying the correlation between historical epidemic cases of hepatitis E and environmental factors (water quality and meteorological data). Environmental factors include many features, and ones that are most relevant to HEV are selected and input into the ensemble learning model composed by Gradient Boosting Decision Tree (GBDT) and Random Forest for training and prediction. Three indicators, root mean square error (RMSE), mean absolute error (MAE) and mean absolute percentage error (MAPE), are used to evaluate the effectiveness of the ensemble learning model against the classical time series prediction model. It is concluded that the ensemble learning model has a better prediction effect than the classical model, and the prediction effectiveness can be improved by exploiting water quality and meteorological factors (radiation, air pressure, precipitation).

## 1. Introduction

According to the World Health Organization (WHO), about one third of the world’s population is exposed to the threat of hepatitis E virus (HEV) and is at risk. About 20 million people are infected with HEV every year. In 2015, there were 44,000 deaths due to HEV infection worldwide, accounting for 3.3% of all viral hepatitis deaths [1]. Hepatitis E has been found in various countries and regions all over the world, but it is more common in developing countries in Asia and Africa, with outbreaks from time to time, and sporadic cases also occur in developed countries [2]. Hepatitis E is an intestinal infectious disease transmitted by fecal oral route. The transmission of hepatitis E can be divided into food borne and water-borne. The incidence of hepatitis E is related to the climate, because climate change impacts the environment, e.g., by affecting the water and food supply. Certain research [3] has already demonstrated the relationship between climate change and bacterial infections. In this work, we want to further explore the relationship between climate change and the incidence of Hepatitis E.

The traditional early warning work of infectious diseases usually adopts the empirical statistical method. By collecting the historical infectious disease case data, the statistical model is used to study the incidence trend of infectious diseases. The time series of EEG (Electroencephalogram) are used to judge the onset of Alzheimer’s disease [4]. Clinical historical influenza incidence data is used to build an early prediction model for influenza like infectious diseases [5]. In 2005, Kulldorff constructed a prospective spatio temporal scanning statistical model and applied it to early detection of disease outbreaks based on case numbers alone. This method does not need the data of the population at risk. It makes the smallest assumptions about the time, geographical location or scale of the outbreak, and adjusts them according to the changes of space and time [6].

With the continuous development of science and technology, researchers began to integrate external factors into the early warnings for infectious diseases, such as economic activities, climate change and population mobility. The WHO has established an early warning system for malaria incidence rate based on monthly case reports and local rainfall in Burundi [7]. In the era of artificial intelligence, disease prediction also has a new method to keep pace with the times. Kathy Lee et al. proposed a real-time influenza and cancer monitoring system based on the space, time and text mining of Twitter data [8]. These studies demonstrate the potential for disease prediction using complex environmental factors. The objective of this study was to predict the incidence rate of hepatitis E by exploiting complex environmental factors with machine learning. The data we used included the historical cases of hepatitis E and the hydrological and meteorological data of the case environment. We used two different machine learning models (GBDT and Random Forest) to improve the accuracy of the prediction model by ensemble learning.

## 2. Method Overview

The traditional early warning for hepatitis E in China usually adopts the empirical statistical method, which only uses historical hepatitis E disease case data to predict the incidence of hepatitis E in the future. In contrast, our approach uses ensemble learning method based on the integration of hepatitis E case data, meteorological data and water quality data to perform prediction and try to achieve an early warning ability.

We obtained hepatitis E cases in a southeast province from 2009 to 2018 and collected the climate, temperature, humidity, water pollution and other related environmental data in this region. After preprocessing, those related environmental data were used for feature selection: to select factors most relevant to hepatitis E.

**Figure 1:**
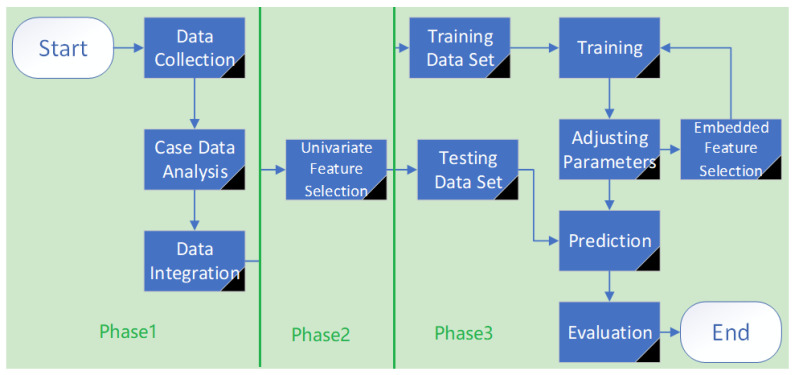
Approach overview.

Our approach is outlined in Figure 1.

Phase 1. Exploring the statistical properties of case data of hepatitis processing abnormal and missing values of case data, water quality data and meteorological data.

Phase 2. Performing correlation analysis and feature selection on the preprocessed data to facilitate the construction of the prediction model.

Phase 3. Building a prediction model of hepatitis E based on ensemble learning, and comparing it with the classic time series model to verify the advantages and disadvantages of an ensemble learning model.

The rest of the paper is organized as follows. Section 2 briefly introduces knowledge from ensemble learning. Section 3 advances the prediction model of hepatitis E based on ensemble learning. Section 4 discusses the evaluation of prediction models and Section 5 discusses the experiments that were performed. Section 6 reviews related work and Section 7 provides conclusions and future work.

## 3. Brief View of Ensemble Learning

We always hope to get accurate and stable one training models. In practice, the training results are not so ideal, as sometimes only a few biased models can be obtained. By calculating the probability of this outcome occurring, we concluded that if there are several independent models, the performance of the combined model is much better than that of a single model. For example, for the classification problem, a trained model’s prediction accuracy rate could be 55%, which is at a medium level. If there are 100 of these types of models, the probability of correct classification results of the ensemble model can increased to 82%. Ensemble learning combines multiple models with medium performance to get a better performance prediction model. The underlying idea is: for the classification problem, although one model gets the wrong classification result, other models can correct the error; for the regression problem, the prediction result of one model has a large error, but the prediction results of other models can balance the error.

Using different machine learning techniques to obtain multiple approximately independent models is not practically operative. Different models have different parameters and different requirements for input data. Therefore, the key to ensemble learning is to generate many approximately independent models. From this we can get a two-tier structure of ensemble learning, and the underlying algorithm is called a base learner. Base learners are relatively independent machine learning algorithms. In the next step, they are integrated into an ensemble learning model. The commonly used base learner is a binary decision tree. The upper level algorithm makes the base learners almost independent of each other, and combines them. Currently, the widely used ensemble methods are bagging and boosting.

The Gradient lifting method is one of the classical boosting algorithms, which uses the decision tree as the base learner and generates a model called a gradient boosting decision tree (GBDT). The decision tree used in GBDT is a regression tree. The goal of each training is to reduce the error of the last training and finally get the minimum error. The model uses the gradient descent method to reduce the error, and the default loss function is the mean square error:

(1) $$L\left(y,f\left(x\right)\right)=\frac{1}{n}\overset{n}{\underset{i=1}{\sum }}{\left(y_{i}-f\left(x_{i}\right)\right)}^{2}$$

where *y* is the measured value and $f\left(x\right)$ is the prediction value. The model is expressed as:

(2) $$F\left(x\right)=\overset{M}{\underset{m=1}{\sum }}\beta_{m}h\left(x;\alpha_{m}\right)$$

where $\beta_{m}h\left(x;\alpha_{m}\right)$ is the m-th base learner,$\;h\left(x;\alpha_{m}\right)$ is the m-th decision tree,$\;\alpha_{m}$ is its parameter and $\beta_{m}$ is the weight in each iteration. Then the optimal prediction function is as follows,

(3) $$F^{*}\left(x\right)=arg\underset{F\left(x\right)}{\min}F_{y,x}\left[L\left(y,f\left(x\right)\right)\right].$$

It can be seen from Equation (2) that to find the optimal prediction function, it is necessary to find the optimal $\left(\beta_{m},\alpha_{m}\right)$ as follows,

(4) $$\hat{\left(\beta_{m},\alpha_{m}\right)}=arg\underset{\alpha_{m},\beta_{m}}{\min}\overset{N}{\underset{i=1}{\sum }}L\left(y_{i},f_{m-1}\left(x_{i}\right)+\beta_{m}h\left(x_{i};\alpha_{m}\right)\right)$$

where $f_{m-1}\left(x_{i}\right)$ is the base learner of the *m* − 1-th training, while $f_{m-1}\left(x_{i}\right)+\beta_{m}h\left(x_{i};\alpha_{m}\right)$ is the base learner of the m-th training.

Given training data $T={\left\{\left(x_{i},y_{i}\right)\right\}}_{i=1}^{N}$, the processing steps of GBDT are:

Initializing the base learner, generating a decision tree with a depth of 1.

(5) $$f_{0}\left(x\right)=arg\underset{c}{\min}\overset{N}{\underset{i=1}{\sum }}L\left(y_{i},c\right)$$

For the *m*-th decision tree:

Calculate the negative gradient, or residual, where i=1,2,⋯,N:

(6) $$r_{im}=-{\left[\frac{\partial L\left(y_{i},f\left(x_{i}\right)\right)}{\partial f\left(x_{i}\right)}\right]}_{f\left(x\right)=f_{m-1}\left(x\right)}.$$

Taking the residuals obtained in (1) as the measured values of new samples, upon which a new decision tree is constructed to obtain the leaf node region: $R_{jm},\;j=1,2,\cdots ,J.$

Calculating the best fitting value of $R_{jm}$:

(7) $$c_{mj}=arg\underset{c}{\min}\underset{x_{i}\in R_{mj}}{\sum }L\left(y_{i},f_{m-1}\left(x_{i}\right)+c\right)$$

which can be updated:

(8) $$f_{m}\left(x\right)=f_{m-1}\left(x\right)+\overset{J}{\underset{j=1}{\sum }}c_{mj}I\;\left(x\in R_{mj}\right)$$

thereby outputting decision tree $f_{M}\left(x\right)$.

## 4. Prediction Model Based on Ensemble Learning

In our approach, GBDT (Gradient Boosting Decision Tree) and random forest were used to construct prediction models for hepatitis E. The process of a hepatitis E prediction model is as follows:

### 4.1. Data Acquisition and Cleaning

The data of hepatitis E cases, population data and regional coding data used in this study are collected from a southeast province in China. Meteorological data is collected from greenhouse data sharing platform. The surface water monitoring data is collected from China’s national environmental monitoring station. The hepatitis E data contain five attributes: gender, age, occupation, region and time of onset. This paper mainly analyzes the reported cases of hepatitis E from 2010 to 2018.

Data cleaning is of great importance. The cleaning methods involved in this paper include abnormal value processing, missing value processing and data coding.

The meteorological data comes from the daily monitoring data of national monitoring stations of the greenhouse data sharing platform. As shown in Table 1, meteorological data includes atmosphere temperature, surface temperature, wind speed, sunshine hours, relative humidity, precipitation, evaporation and station air pressure. Some meteorological data is missing due to detection equipment or data storage malfunction. Hence, cleaning meteorological data deals with abnormal and missing values.

**Figure 2:**
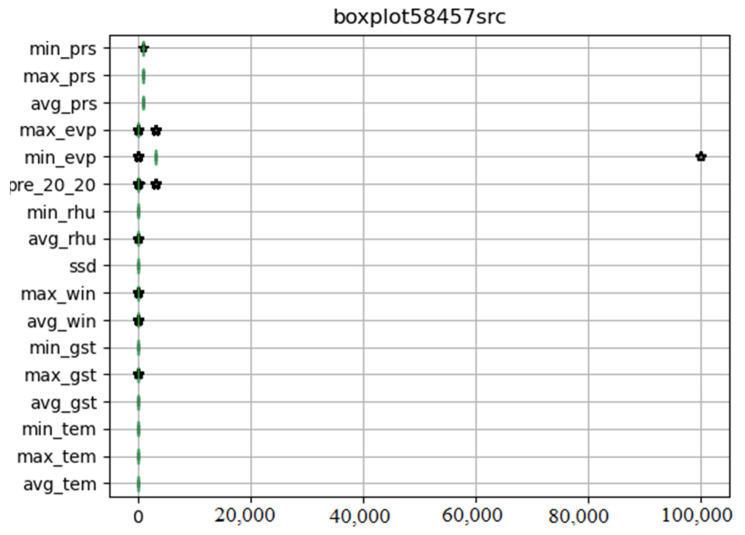
Illustration of the original meteorological data with abnormal data.

**Figure 3:**
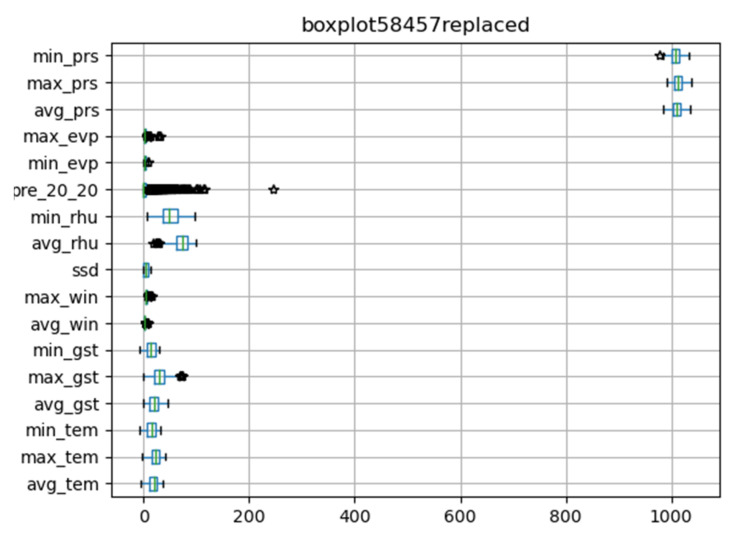
Illustration of the meteorological data after cleaning for abnormal values.

We drew the box diagram with Python *matlibplot.pyplot*, and checked the abnormal value of meteorological data with the *describe* function of *dataframe* class in *pandas* package. Figure 2 shows original meteorological data obtained from 58,457 meteorological stations. The abnormal values are special codes in meteorological data. They were replaced by pandas. Dataframe.replace function. Figure 3 shows meteorological data after processing.

After that, in line with our target, we performed feature construction to generate more sophisticated features to better describe some more complex phenomena, based on the meteorological data and water quality data after processing and the incubation period of hepatitis E. Some of the constructed features are illustrated in Table 2.

The data of hepatitis E were then sorted into time series data. Finally, the water quality characteristics, meteorological characteristics and the time series of hepatitis E cases were integrated.

### 4.2. Feature Selection

Feature selection determines whether the selected features are useful for the prediction target. It is necessary to understand some background knowledge related to the problem when constructing the feature. The general principle is to use one’s imagination to create features as often as possible. If the new features contain redundant features, then the redundant features can be deleted through feature selection. Feature design and selection need iterative verification to get better results. After constructing features according to the relevant domain knowledge, the correlation coefficient method is used to select features, and then the embedding method is used to further select features in the subsequent modeling process.

Feature selection refers to selecting a feature subset from the complete feature set which is beneficial to the training model and is as small as possible. An appropriate feature subset can reduce the running time of the model, improve the performance of the model and enhance the interpretability of the model. A practical problem often has many attributes (i.e., features), but these attributes are not related to the problem; usually there are some irrelevant features and redundant features that interfere with the construction of the model. Therefore, we needed to filter the feature set to reduce the difficulty of the model, and improve its performance.

This paper used the combination of a filtering method and embedding method for feature selection. Firstly, the univariate feature selection method was used to filter out the features with low correlation with the prediction target. Then, the embedding method was used to further screen features in the follow-up experiments.

Feature selection based on the filtering method is included in data preprocessing, before model training. The method evaluates the correlation between different features and prediction results by calculating the statistical characteristics or information entropy of each feature. The feature subset which is beneficial to model training was selected. This method is based on single variable analysis with the correlation coefficient method.

Feature selection based on the embedding method directly takes feature selection as a part of a machine learning model. Some machine learning models can score features themselves. We can use these models to train, obtain the importance ranking of input features and select features according to their importance.

### 4.3. Parameter Adjustment

The preliminary selected features were used as the input of GBDT/random forest. The model parameters were adjusted, and the features are screened and trained. Finally, the test set was used to predict and analyze the results.

The adjustment of ensemble learning parameters starts from the base learner and the upper level algorithm. The GBDT and random forest are both decision trees, so the parameters that need to be adjusted in the decision tree should be considered first. max_ Depth sets the maximum depth of the tree; a deeper decision tree may get better prediction results in the random forest, but a deeper tree needs a longer training time and incurs a risk of over fitting. We needed to balance these pros and cons. In the gradient lifting method, the iterative process will correct the wrong results, hence the shallow decision tree can get good results.

In the adjustment of the upper level algorithm, both GBDT and random forest should set n_estimators, which specifies the number of base learners in the ensemble method. In the algorithm based on a decision tree, n_estimators and the maximum depth of the tree jointly determine the complexity of the model. If the parameters are too small, this will cause over fitting of the model, and if they are too big, it will lead to the model not fitting well. Many attempts are needed to determine the best values. In GBDT, the learning_Rate also needs to be set. When the learning rate is too large, the training error will drop rapidly, which is likely to cause over fitting. If the learning rate is too small, then more iterations are needed to obtain better results, which will reduce the training speed. n_Estimators and learning_ Rate will affect each other and are usually adjusted together. In addition, these two values will also affect the choice of optimal maximum depth.

## 5. Evaluation Metrics

**Figure 4:**
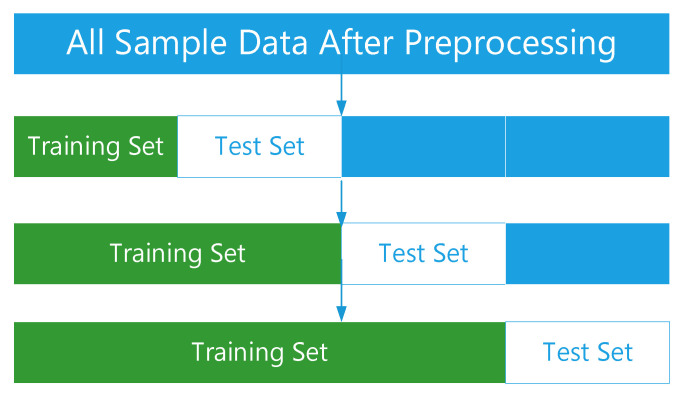
Evaluation Model.

Cross validation is a commonly used verification method in model optimization and parameter adjustment. It improves the generalization ability of the model by evaluating the parameter setting and prediction effectiveness. We adopted a three folded time series cross validation, as seen in Figure 4.

*Mean absolute error* (MAE): The mean absolute error is the mean absolute error of all samples.

(9) $$\mathrm{MAE}=\frac{1}{n}\overset{n}{\underset{i=1}{\sum }}\left|y_{i}^{\prime }-y_{i}\right|$$

where $y_{i}^{\prime }$ is prediction value of a sample, $y_{i}$ is measured value, MAE is ranged over $\left[0,\;\left.+\infty \right)\right..$

*Mean absolute percentage error* (MAPE): MAPE is the mean absolute percentage error of all samples.

(10) $$\mathrm{MAPE}=\frac{100\%}{n}\overset{n}{\underset{i=1}{\sum }}\left|\frac{y_{i}^{\prime }-y_{i}}{y_{i}}\right|$$

where $y_{i}^{\prime }$ is the prediction value,$\;y_{i}$ is the measured value and MAPE is ranged over $\left[0,\;\left.+\infty \right)\right..$ When the predicted value is the same as the measured value, MAPE is 0. If MAPE is greater than 1, it means that the model is of poor quality. Compared with MAE, MAPE is more intuitive to express the quality of the model, but it should be noted that when the measured value is 0, MAPE cannot be used.

Root mean square error (RMSE):

(11) $$\mathrm{RMSE}=\sqrt{\frac{1}{n}\sum_{i-1}^{n}{\left(y_{i}^{\prime }-y_{i}\right)}^{2}}.$$

The time series cross validation method was used to evaluate the prediction effect of the model. The experimental data are divided into three groups of training sets and test sets by using the fold time series cross validation method. This method can reflect the generalization ability of the model and the laws contained in the data of different periods and time spans. The influence of different scale training sets on the prediction effect can be found through it. The RMSE, MAE and MAPE of the model on the test set were calculated to evaluate the prediction effect of the model.

## 6. Experiments

### 6.1. Experiments Overview

This section discusses implementation of the prediction model of hepatitis E based on ensemble learning, specifically using GBDT and random forest two ensemble methods, and comparing this approach with the classic time series model of the infection prediction method. We selected the model with better prediction effect, combined with the moving percentile method and the moving average method to provide early warning of the high incidence of hepatitis E.

Experiment 1: implementing the prediction model of the monthly incidence of hepatitis E based on GBDT/random forest.

Experiment 2: implementing the prediction model of monthly incidence of hepatitis E based on the time series method, and comparing the performance of three prediction models;

Experiment 3: Using the ensemble learning model combined with the moving percentile method and the moving average method to predict the high incidence month of hepatitis E in 2018.

### 6.2. Experiments of Ensemble Learning Models

The data of hepatitis E cases, population data and regional coding data used in this study were collected from a southeast province in China. Meteorological data was collected from a greenhouse data sharing platform. The surface water monitoring data was collected from China’s national environmental monitoring station. The data of hepatitis E contains five attributes: gender, age, occupation, region and time of onset. This paper mainly analyzes the reported cases of hepatitis E from 2010 to 2018. Based on the data of hepatitis E cases, water quality and meteorological factors from 2010 to 2018, we divided the training set and test set by using the three-fold time series cross validation method, and used RMSE, MAE and MAPE as evaluation indexes to train the GDBT model and random forest model to determine their optimal parameters.

#### 6.2.1. GBDT Experiments

Mean square error and absolute error synthesis were selected as the loss function of the GBDT model. That is, the loss parameter was set to “Huber”, and several important parameters of the model were adjusted respectively to select their optimal combination.

First *n_estimators* was set to 1000 and other parameters were set to default values. The mean square error of the training set and the test set initially decreased very quickly, while the mean square error of the test set began to rise after the decline. The model was over fitted. We therefore reduced the step size of gradient descent and adjusted the relevant parameters of the decision tree. We compared the performance of different decision tree depths to select the appropriate decision tree depth. Through experiments, the tree with depth 1 was selected as the base learner. The final parameters are shown in Table 3.

The above parameters were used to train the three groups of data. The fitting degree of the three groups of training sets is shown in Table 4.

Test set prediction performance is shown in Table 5.

**Figure 5:**
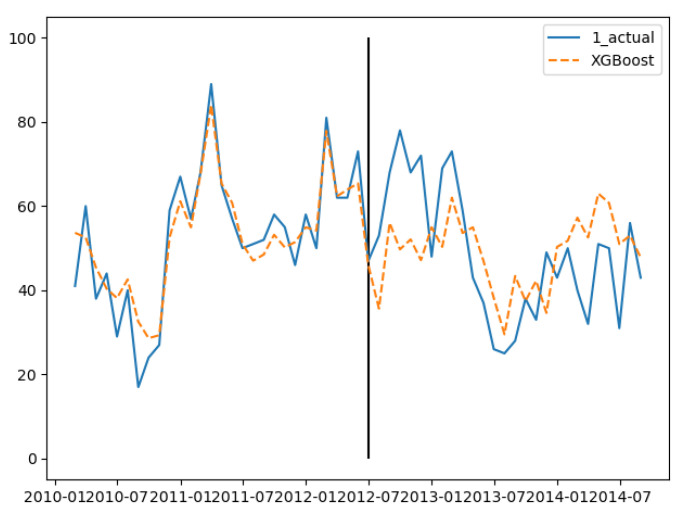
GBDT’s fitting and prediction results for group 1.

**Figure 6:**
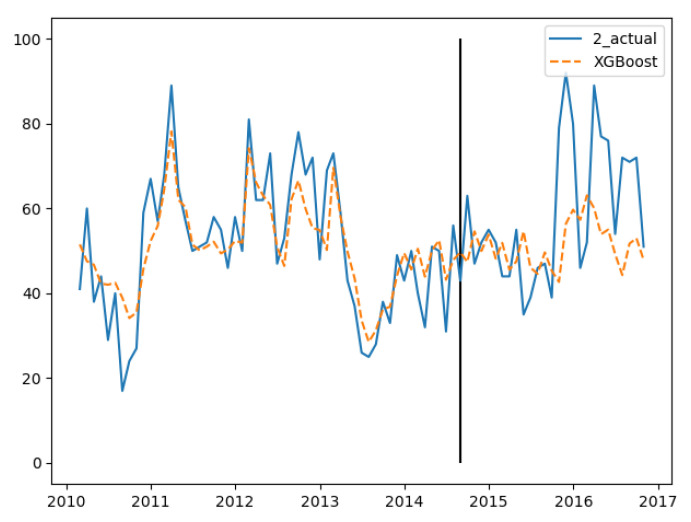
GBDT’s fitting and prediction results for group 2.

**Figure 7:**
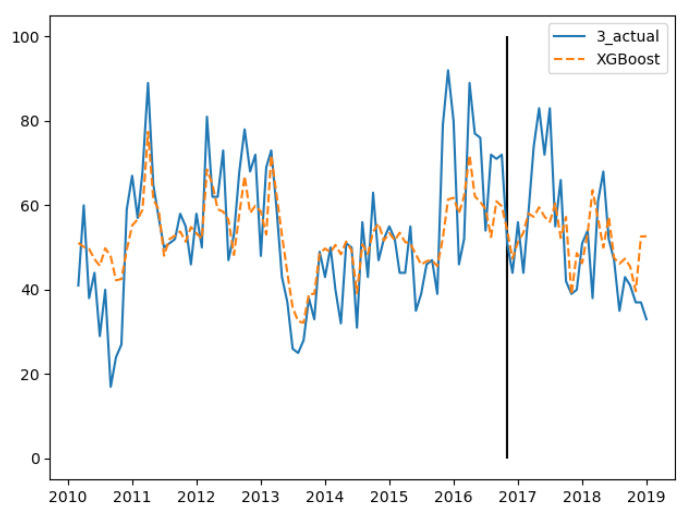
GBDT’s fitting and prediction results for group 3.

In the three groups of experiments, the smaller the training set, the better the fitting degree. The prediction results of the third group were the best and the second group were the worst. The fitting and prediction results of three groups of data are shown in Figure 5, Figure 6 and Figure 7, respectively. It can be seen from the figures that the number of prediction cases in group 2’s testing set was abnormally convex for a period. The fitting degree in the same period in the group 3’s training set was also poor. The reason for this is that the transmission factors of infectious diseases are complex, the reasons for which are not covered in this paper.

In viewing the features of the GBDT model, six features, ‘the number of patients in the last month’, ‘the average daily minimum pressure in the first two months’, ‘the average potassium permanganate index in the first three months’, ‘the average daily maximum wind speed in the first three months’ and ‘the average daily radiation in the first three months’, were screened out.

#### 6.2.2. Random Forest Experiments

Mean square error is the loss function of the Random Forrest model. Parameters were set as shown in Table 6.

The above parameters were used to train the three groups of data. The fitting degree of the three groups of training sets is shown in Table 7. Test set prediction performance is shown in Table 8.

**Figure 8:**
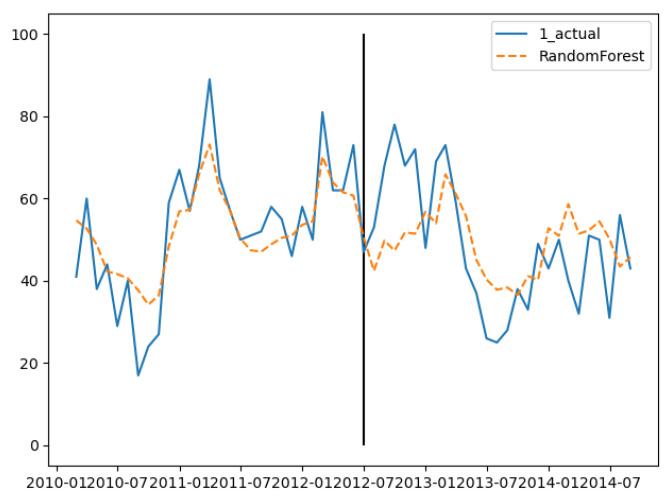
Random forest’s fitting and prediction results for group 1.

**Figure 9:**
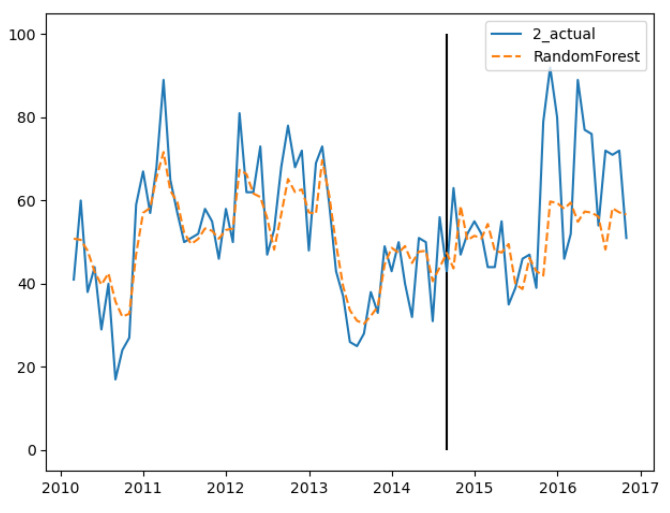
Random forest’s fitting and prediction results for group 2.

**Figure 10:**
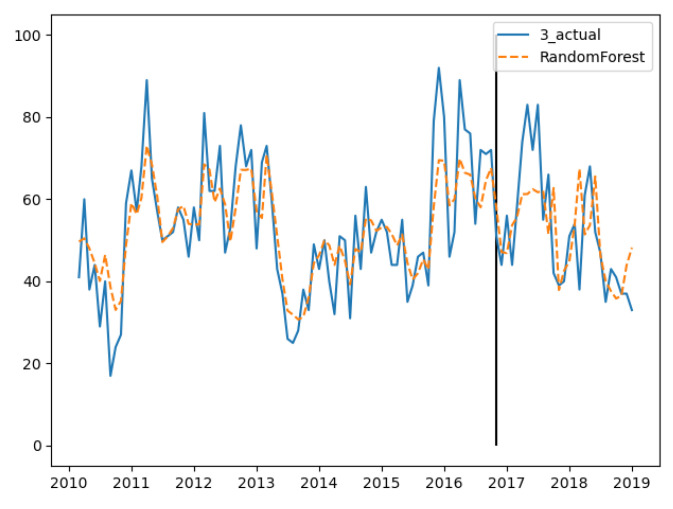
Random forest’s fitting and prediction results for group 3.

In the training set, the three groups’ fitting degree showed little difference. In test set, the third group has the best prediction performance. Figure 8, Figure 9 and Figure 10 show the fitting and prediction results of random forest. In the second and third groups of experiments, the model could predict the increase of the number of cases, but there was still a certain gap in the numerical value. In general, the prediction results of random forest were slightly better than GBDT.

According to the feature importance score, five features, (the number of patients in the last month), (the average potassium permanganate index in the first three months), (the average daily minimum ground temperature in the first two months), (the average daily maximum wind speed in the first three months and the average precipitation in the first two months) were finally screened out.

### 6.3. Comparative Experiment

The time series prediction model has been commonly used in disease prediction. In this work, SPSS time series statistics software was used to predict hepatitis E cases. Time series cross validation was used to product three training and test sets. Their fitting and predicting processes are analyzed as follows.

**Figure 11:**
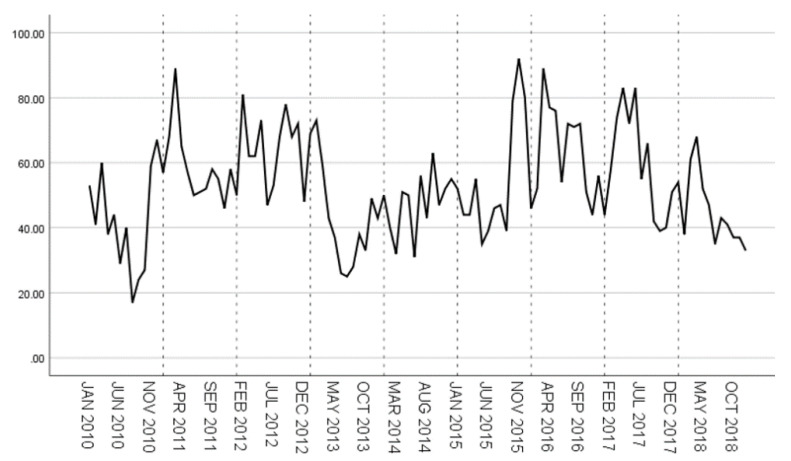
Original time series of hepatitis E from 2010 to 2018.

The original time series of hepatitis E case data from 2010 to 2018 is shown in Figure 11. It can be seen that from 2010 to 2018, the number of hepatitis E cases has no obvious upward or downward trend. Except for 2012, 2014 and 2015, the incidence of hepatitis E was generally high in winter and spring.

Based on the above analysis, the simple seasonal model of exponential smoothing method was used to fit the three groups of data. The exponential smoothing method is a time series analysis and prediction method developed on the basis of the moving average method. It is used to predict the future of a phenomenon by calculating the exponential smoothing value and combining it with a certain time series prediction model. The principle is that the exponential smoothing value of any period is the weighted average of the actual observed value and the exponential smoothing value of the previous period. In the exponential smoothing method, the simple seasonal model is especially suitable for a time series with periodic characteristics because of considering seasonal effects. We used the SPSS to conduct the fitting of the simple seasonal model for the prediction of hepatitis E.

The average RMSE was 11.42, MAE was 8.807 and MAPE was 17.91%, The fitting degree of each training set is shown in Table 9. RMSE, MAE and MAPE showed that the model fitting degree of the first training set was the best. Table 10 shows the prediction performance comparison of the three test sets.

**Figure 12:**
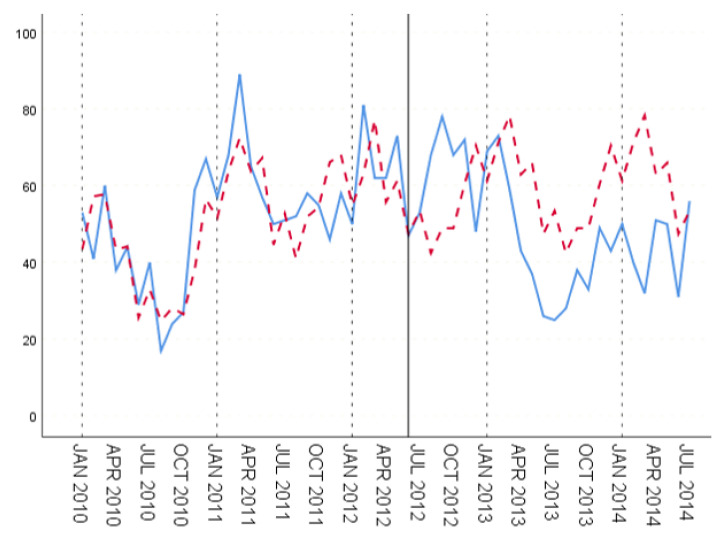
Fitting and prediction results for the time series model, group 1.

**Figure 13:**
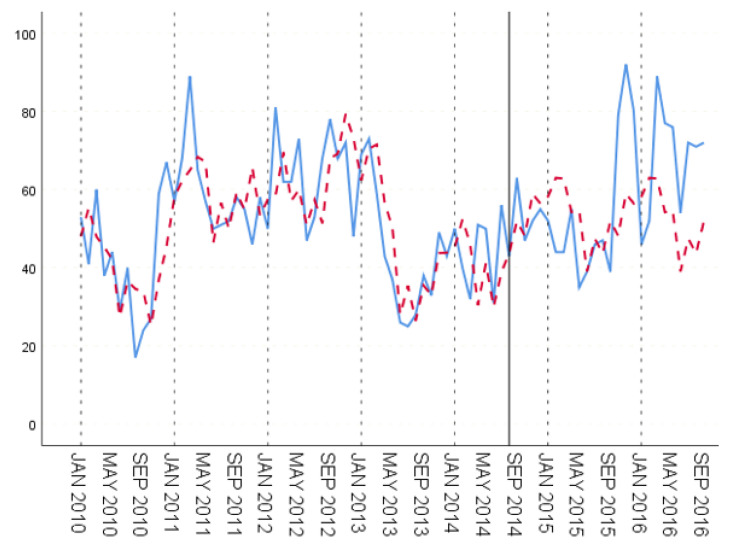
Fitting and prediction results for the time series model, group 2.

**Figure 14:**
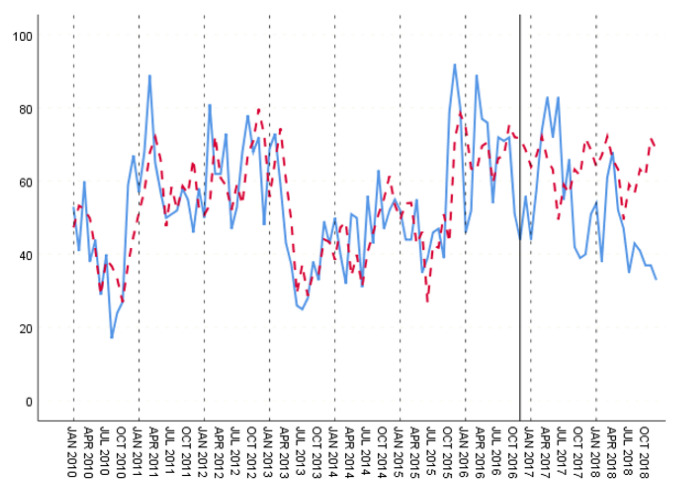
Fitting and prediction results for the time series model, group 3.

Figure 12, Figure 13 and Figure 14 show the fitting results of training set and prediction result of test set, respectively. In the three groups of experiments, according to the evaluation index, the second group has the best prediction effect, that is to say, the relatively good prediction effect can be obtained by training the historical case data for about five years.

The prediction effect of time series model for the first two periods of the test set is obviously better than that of the later period, which is more obvious in the first and second groups of data, namely, the medium and short-term experiments. This is a feature of the time series model, where prediction depends on historical data. The further ahead the time of the prediction data is from the evaluation period, the worse the prediction effect is.

The experiment data of the three groups shows that after adding meteorological factors and water quality factors, compared with the time series model, the prediction effect of hepatitis E monthly incidence based on the ensemble learning model is improved. The RMSE, MAE and MAPE were used to compare the time series model with the two ensemble learning models in Table 11. The prediction effect of ensemble learning is better than that of the time series model. Among them, random forest is slightly better than GBDT.

According to the comparison, we could conclude that our method could largely improve the prediction accuracy. The traditional empirical statistical method only relies on case reports, which can typically suffer from delay issues. Our method based on the combination of history case data with other data, such as meteorological data and water quality data, would provide a more timely approach.

In this section, the fitting prediction results of time series model and ensemble learning model for hepatitis are compared. In the experiment involving the ensemble learning model, we selected the features for prediction. Experiments show that ensemble learning is better than the time series model, and the prediction effect of random forest is slightly better than that of GBDT.

## 7. Discussion

To our knowledge, this study is the first attempt to use the ensemble learning model based on the integration of hepatitis E case data, meteorological data and water quality data to perform prediction and try to achieve an early warning ability.

Prediction and early warning of diseases, especially for infectious disease, is of great importance. Relying on the rapid development of statistics, computer science and other disciplines, the United States pioneered the research and exploration of modern infectious disease early warning technology. In 1924, Walter A. Shewhart proposed the control chart method, which was later applied to the national disease surveillance system by the United States Department of Health [9]. In the 1970s, Box and Jenkins put forward the time series prediction method. This method can be used to understand the long-term development trend of things by analyzing the historical changes of things and making predictions. It is still widely used in the prediction of various diseases [10]. In 1998, Kleinman et al. proposed a generalized linear mixed model, and used this model to provide an early-warning for infectious diseases caused by bio-terrorism in a small area [11].

Other countries have quickly joined the trend. In 2003, Australia launched an automated real-time public health monitoring system during the Rugby World Cup. Based on the data collected by the emergency system, the system uses text classification technology to monitor epidemic situations in Sydney in real time [12]. In 1997, Canada and who jointly established the global public health intelligence network to monitor global epidemic information by identifying Internet information such as news and blogs [13].

In 2003, severe acute respiratory syndrome (SARS) broke out in China. The Chinese government realized that a simple reporting system for infectious diseases has been unable to support the needs of the prevention and control of infectious diseases in modern society. Then, the network direct reporting system of infectious diseases and public health emergencies was quickly established, which changed the reporting and management of epidemic information fundamentally [14]. In 2008, based on the network direct reporting system, China built an automatic infectious disease prediction system. The early warning function for an epidemic outbreak was realized by using the historical monitoring data and spatial temporal model of infectious diseases [15]. In 1982, Professor Deng Julong established the gray model. This model can reveal the continuous development and change process of things in the case of incomplete information. It is widely used in the prediction of infectious diseases. Chinese scholars have applied it to the prediction of intestinal infectious diseases, insect borne infectious diseases, sexually transmitted diseases and other infectious diseases [16,17,18]. Zeng Guang, Ding Yanpeng and Cheng Yingkai used retrospective methods to analyze the historical monitoring data of various notifiable infectious diseases in China. The study found that if the seasonal incidence curve of a contagious disease showed a right (left) deviation, then the incidence rate of the next cycle tends to have an increase (decrease). The more the deviation, the greater the probability of an increase or decrease in incidence rate. This phenomenon is named “Zeng-Ding” [19]. Subsequently, more scholars have studied the verification and prediction of “Z-D” phenomenon in a variety of infectious diseases [20,21]. In 2002, Guo Xiuhua and other scholars proposed to predict seasonal series by wavelet analysis. Based on the historical data of hemorrhagic fever from 1995 to 1998, a model was established to predict its incidence rate in 1999 in Heihe and Linyi [22]. Based on the research results of foreign scholars on early warning of infectious diseases, control chart method and time series model are also widely used in the research of infectious disease prediction and early warning in China [23,24].

Research on complex network theory finds that human society is regarded as a network model with uneven distribution, in which there are obvious scale-free effects and small world effects. Each individual and other individuals in the social network show aggregation, which coincides with the epidemic characteristics of infectious diseases. Therefore, the spread of infectious diseases in the community can be regarded as the transmission behavior on complex networks. In order to prevent and control infectious diseases, the complex network predicts its transmission mechanism and finds out the key nodes to control and isolate them [25]. In 2006, Gong Jianhua and others established the SARS dynamic transmission model, and used multi-agent technology to construct the individual based spatiotemporal transmission mechanism of SARS [26].

Machine learning methods and artificial intelligence methods are now very popular. Much research has been trying to use those methods in the public health domain. An artificial neural network can automatically adjust the structure of the model to adapt to the characteristics of the things analyzed, and learn the rules between the features, which is suitable for the research on infectious disease prediction [27]. Google influenza trends (GFT) uses anonymous, aggregated Internet search activities to provide real-time estimates of influenza activity. It performed well at the time of the global outbreak of influenza A (H1N1) in 2009 [28]; however, it was discontiuned due to important errors.

So, in this work, we have presented our effort to use the ensemble learning method to implement prediction and early warning systems for hepatitis E.

## 8. Conclusions and Future Work

Hepatitis E is an intestinal infectious disease transmitted by a fecal oral route. The outbreaks of hepatitis E in China can be divided into two categories: food borne and water-borne. In most areas, the incidence of hepatitis E is high in spring. This is because climate change has impacts on the weather and environment in a certain area, including on its water and food.

The outbreak of infectious diseases often involves many factors, including society, economy, population and so on. It is very complex to forecast and provide early warnings of infectious diseases accurately.

In this paper, the incidence of hepatitis E was predicted using an ensemble learning model, which was trained with case data, meteorological data and water quality data. Specifically, the ensemble learning method was applied to the prediction and early warning of hepatitis E, and the moving percentile method was applied to the early warning of hepatitis E. In addition, the meteorological factors and water quality factors that may affect the incidence of hepatitis E were integrated into the prediction of hepatitis E.

According to the results, our method generates a much more accurate prediction result compared to the traditional empirical method. Traditional empirical statistical methods such as the time series model relies only on the case reports of hepatitis E, which would typically suffer from a delay issue. In our method, we used an ensemble learning method to implement a prediction approach based on the integration of hepatitis E case data, meteorological data and water quality data. The involvement of meteorological data and water quality data could largely ease the dependence on timely reports. Also, our way could provide a way to achieve early warning of hepatitis E outbreaks, since hepatitis E’s transmission is clearly related to weather and water.

There are still many areas to be improved in the research of prediction and early warning methods for hepatitis E. The following aspects may be explored:

Applying other machine learning algorithms to predict the incidence trend of hepatitis E and further improving the accuracy of predictions.

Hepatitis E is a food borne infectious disease, while the epidemic characteristics of different regions have major differences between them. Due to the gradual improvement of the Chinese national health level, the association between hepatitis E and living environment may be weakened. Its transmission involves population, economic and other factors, so it is necessary to consider the relationship between these other factors and hepatitis E.

## Figures and Tables

**Table 1:** Original meteorological data.

Attribute Name	Meaning	Unit
avg_tem	Average temp	°C
max_tem	Daily maximum temperature	°C
min_tem	Daily minimum temperature	°C
avg_gst	Mean surface temperature	°C
max_gst	Daily maximum surface temperature	°C
min_gst	Daily minimum surface temperature	°C
avg_win	Average wind speed	m/s
max_win	Maximum wind speed	m/s
ssd	Sunshine hours	h
avg_rhu	Average relative humidity	%
avg_rhu	Minimum relative humidity	%
pre_20_20	Accumulated precipitation from 20:00 to 20:00	mm
min_evp	Small evaporation	mm
max_evp	Large evaporation	mm
avg_prs	Average local air pressure	hPa
max_prs	Daily maximum local air pressure	hPa
min_prs	Daily minimum local air pressure	hPa

**Table 2:** Meteorological and water quality features.

Attribute	Meaning	Unit
avg_tem_2m	Average temperature of the first two months	(°C)
avg_tem_3m	Average temperature of the first three months	(°C)
avg_win_2m	Average wind speed in the first two months	(m/s)
pre_sum_2m	Accumulated precipitation days in the first two months	(d)
do_2m	Average dissolved oxygen in the first two months	mg/L
nh3_n_2m	Average high ammonia nitrogen in the first two months	mg/L
qual_2m	Water quality category in the first two months	1\2\3\4\5\6

**Table 3:** Gradient boosting decision tree (GBDT) Parameter Settings.

Parameter Name	Meaning	Value
loss	Loss function	huber
n_estimators	Number of decision tree	300
max_depth	Maximum depth	1
learning_rate	Step size of gradient lifting	0.02
max_features	The number of features	sqrt

**Table 4:** GBDT Fitting degree under a training set.

	RMSE	MAE	MAPE
Group1	5.767	4.616	11.85%
Group2	8.337	6.807	16.17%
Group3	10.743	8.478	18.55%
Average	8.282	6.634	15.53%

**Table 5:** GBDT prediction performance under a test set.

	RMSE	MAE	MAPE
Group1	13.961	12.150	26.83%
Group2	17.001	13.291	20.82%
Group3	12.816	9.982	19.80%
Average	14.593	11.807	22.48%

**Table 6:** Random forest parameter settings.

Parameter Name	Meaning	Value
n_estimators	Number of decision trees	200
max_depth	Maximum depth of trees	5
min_samples_split	Minimum number of samples for node re segmentation	10

**Table 7:** Random forest fitting degree under the training set.

	RMSE	MAE	MAPE
Group1	8.531	6.703	16.95%
Group2	7.859	6.511	15.08%
Group3	8.489	6.880	14.59%
Average	8.293	6.698	15.54%

**Table 8:** Random forest prediction performance under the test set.

	RMSE	MAE	MAPE
Group1	13.379	11.343	25.66%
Group2	16.310	12.606	19.56%
Group3	12.108	9.526	18.65%
Average	13.932	11.159	21.29%

**Table 9:** Fitting degree of the training set of the time series model.

	RMSE	MAE	MAPE
Group1	10.455	8.158	16.07%
Group2	11.324	8.612	18.36%
Group3	12.482	9.650	19.29%

**Table 10:** Prediction performance of test set of time series model.

	RMSE	MAE	MAPE
Group1	20.274	17.269	42.25%
Group2	17.571	14.269	23.20%
Group3	20.485	17.692	39.92%

**Table 11:** Comparison of the model prediction effect.

	RMSE	MAE	MAPE
Time series model	19.443	16.41	35.12%
GBDT	14.593	11.807	22.48%
Random Forest	13.932	11.159	21.29%

## Data Availability

Requests for data (6/12 months after publication of this article) will be considered by the corresponding author.
